# Global and local oscillatory entrainment of visual behavior across retinotopic space

**DOI:** 10.1038/srep25132

**Published:** 2016-04-29

**Authors:** Rodika Sokoliuk, Rufin VanRullen

**Affiliations:** 1 Université de Toulouse, Centre de Recherche Cerveau et Cognition, Université Paul Sabatier, Toulouse, France; 2 CNRS, UMR 5549, Faculté de Médecine de Purpan, Toulouse, France; 3 School of Psychology, University of Birmingham, Birmingham B15 2TT, UK

## Abstract

Ongoing brain oscillations (7–10 Hz) modulate visual perception; in particular, their precise phase can predict target perception. Here, we employ this phase-dependence of perception in a psychophysical experiment to track spatial properties of entrained oscillations of visual perception across the visual field. Is this entrainment local, or a more global phenomenon? If the latter, does oscillatory phase synchronize over space, or vary with increasing distance from the oscillatory source? We presented a disc stimulus in the upper left quadrant, oscillating in luminance at different frequencies (individual alpha frequency (IAF), 5 Hz, and 15 Hz) to entrain an oscillation with specific frequency and spatial origin. Observers fixated centrally, while flash stimuli at perceptual threshold appeared at different positions and times with respect to the oscillating stimulus. IAF and 5 Hz luminance oscillations modulated detection performance at all tested positions, whereas at 15 Hz, the effect was weaker and less consistent. Furthermore, for IAF and 5 Hz entrainment, preferred phases for target detection differed significantly between spatial locations, suggesting “local” entrainment of detection performance next to the oscillatory source, whereas more distant target locations shared a “global” effect with a significantly different phase. This unexpected global component of entrainment is tentatively attributed to widespread connectivity from thalamic nuclei such as the pulvinar.

Recent studies showed that human visual perception is altered by ongoing brain activity^1^,^2^,^3^,^4^, particularly by oscillations in the alpha and theta frequency bands (7–10 Hz)^5^,^6^,^7^,^8^. However, how exactly this oscillatory activity acts at the anatomical level over the retinotopically organized visual cortex remains unclear. Are oscillations spatially restricted, or broadly distributed? Do oscillations synchronize in phase over space or does their phase change with their spatial distance from the oscillatory source?

Brain oscillations, sustained by an independent area with widespread anatomical connections (e.g., the thalamus), may spread with perfect phase-synchrony over large distances^9^,^10^, possibly encompassing entire retinotopic visual areas. Contrarily, because of the pattern of lateral connectivity in retinotopic visual areas, oscillatory fluctuations in visual activity may be expected to propagate across space like a travelling pulse^11^ or oscillatory wave, respectively^12^,^13^,^14^, with a constant phase shift reflecting the speed of lateral connections (approximately 0.2 to 0.5 m/s)^15^,^16^,^17^,^18^,^11^. Although retinotopic oscillatory travelling waves have been described in visual areas of other species^19^,^20^,^12^,^13^,^14^ (see, however^21^, for conflicting findings), no corresponding evidence exists in humans. Finally, a local oscillation may simply dwindle at a moderate distance from its source, thus limiting the possibility of observing wave-like phase propagation, global phase synchrony or other global phase patterns.

Here, we directly addressed this question in a psychophysical experiment. Participants fixated a central dot while a peripheral disc was oscillating in luminance, in order to entrain oscillations with a specific spatial origin. We first chose to stimulate at the individual alpha frequency as it is known to maximize oscillatory entrainment^22^. Meanwhile, participants were instructed to report via button press the detection of a near-threshold target that could appear at 9 (Experiment 1; see Fig. 1B) or 5 (Experiment 2; see Fig. 1C) possible locations on the screen. Multiple preceding studies showed that visual perception oscillates along with the phase of ongoing brain oscillations in the theta and alpha range (7–10 Hz), revealing a ‘preferred’ phase where detection performance is maximal^5^,^6^,^7^,^8^. Because of this phase dependence of perception, several recent studies have demonstrated that it is possible to modulate perception rhythmically, using entraining sequences at alpha frequency^23^,^24^.We used this property to estimate the phase of the entrained oscillations at each position across the visual field. For each target location, we expressed detection performance as a function of the phase of the entraining luminance sequence at target onset. These functions revealed a clear ‘preferred phase’ at alpha frequency for target detection at all tested positions. Importantly, this preferred phase significantly differed between the target location adjacent to the disc and all other target locations. We then investigated whether luminance oscillations at other frequencies (5 and 15 Hz in Experiments 3 and 4, respectively) would lead to similar results. Both frequencies modulated detection performance in an oscillatory way over the visual field; however, the 15 Hz modulation was much reduced relative to the 5 Hz and alpha modulations. A control experiment revealed that the disc luminance at the moment of target presentation contributed strongly to the oscillatory pattern of detection performance; however, this contribution was locally restricted to target positions near the peripheral disc. We thus hypothesize that our results reflect the co-occurrence of two distinct effects, a *local* effect induced by the oscillating disc’s luminance and a *global* effect reaching more distant target positions and presenting a significantly different phase.

## Results

### Alpha oscillatory entrainment of detection performance across the visual field

Detection performance was calculated separately for each participant and position as a percentage of correct trials (hit rate) over 8 possible phase values of the entrained alpha oscillation (Fig. 2, in blue). In order to reveal the oscillatory character of detection performance over phases, these data were averaged over subjects and fitted to a cosine function (Fig. 2, orange dashed curve). Detection performance showed significant oscillatory amplitudes for all 9 positions across the visual field (Monte Carlo test, p < 0.05, FDR corrected; Fig. 2). The average amplitude of oscillatory entrainment of detection performance (difference between fitted performance at the maximal phase vs. the opposite phase) was 10 ± 1.2% (average ± standard error of the mean (sem) over positions). Detection performance was thus entrained by the alpha-frequency luminance oscillation in the peripheral disc, with a clear detection peak for one of the oscillatory phases.

### Preferred alpha phase for detection performance across the visual field

**Figure 1:**
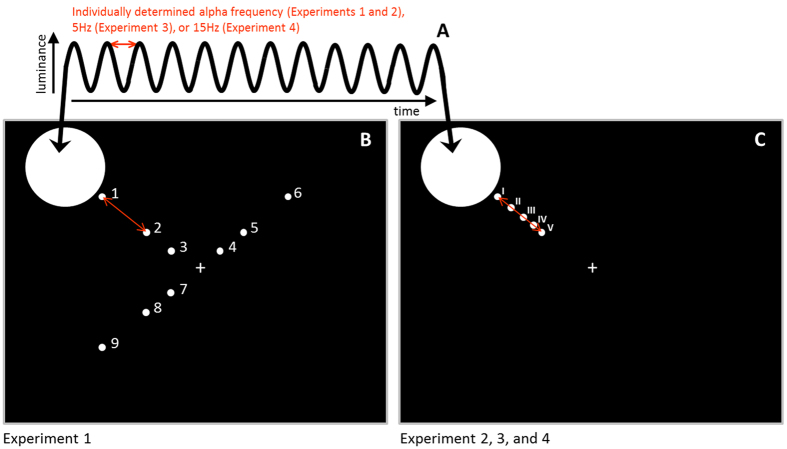
Experimental Design. (**A**) The individual alpha frequency (IAF) of each subject was determined according to resting-state EEG data recorded in a prior experiment. It was used as the frequency of luminance flicker for Experiments 1 and 2. Experiments 3 and 4 used flicker at 5 Hz and 15 Hz, respectively. (**B**) Experiment 1: In each trial (6.25 s), a peripheral disc oscillated sinusoidally at the IAF. Subjects fixated the central cross and reported via button press the detection of near-threshold targets which could appear at 9 possible locations (2–4 single targets were presented over 6.25 s in each trial). Target locations were spaced according to cortical magnification to ensure equal spacing in the visual cortex (here: drawn to scale). In order to keep detection performance at ~50%, target luminance was adapted using a staircase procedure. (**C**) In Experiment 2, participants performed the same task as in Experiment 1 but only 5 locations were tested with a higher spatial resolution (target locations 1 and 2 of Experiment 1 as well as 3 additional locations in-between; drawn to scale). In Experiments 3 and 4, the IAF oscillation was replaced by a 5 Hz and 15 Hz oscillation (respectively). A fifth experiment served as a masking control where detection performance at the same 5 target locations was tested but without oscillatory entrainment of visual detection performance. Here, the peripheral disc was flashed only once, at different SOAs before or after the target (±50, 37.5, 25, 12.5 and 0 ms). Finally, a last control experiment explored the influence of the disc’s luminance: a single flash of the peripheral disc occurred simultaneously with the target (0 ms SOA), but with variable luminance values (4 possible values).

**Figure 2:**
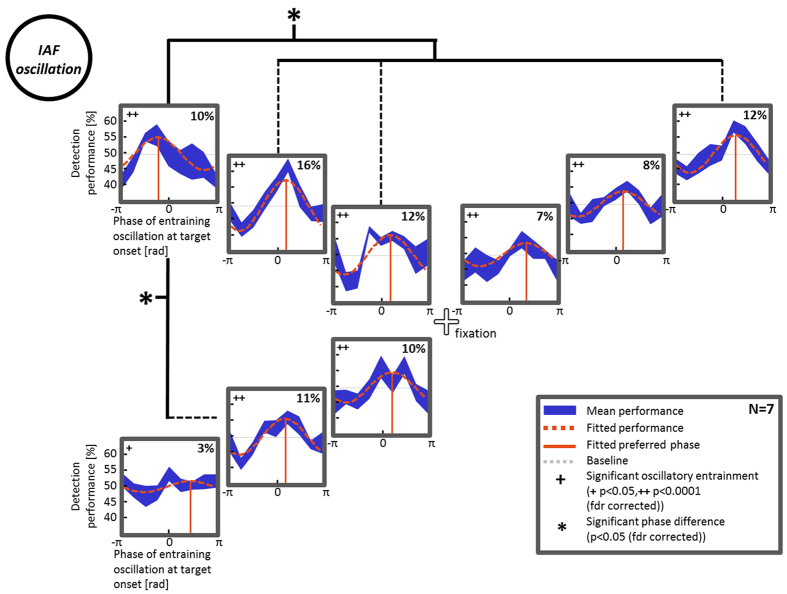
Detection performance oscillates over the visual field with significant phase differences between target locations. Results of Experiment 1 are represented spatially at the 9 possible target locations (not drawn to scale). Mean data of all 7 subjects (in blue, the outer edges represent mean ± sem) were fitted to a cosine function (orange dashed line) to test the oscillatory character of detection performance. A Monte Carlo test revealed significant amplitude of oscillatory modulation of detection performance at individual alpha frequency (IAF) at all 9 positions (values of oscillatory amplitude modulation in the upper right corner of each plot). The phase value of the oscillating disc (oscillatory focus) yielding maximal detection performance (marked by the vertical orange line in each plot) shows significant differences between position 1 (next to the oscillatory focus) and 4 other positions.

For all possible target locations, we computed the phase of oscillatory entrainment (i.e. the phase of the peripheral luminance fluctuation for which target detection performance was maximal). Comparing these ‘preferred phases’ showed significant phase differences (Monte-Carlo test, p < 0.05, FDR corrected; Fig. 2) between position 1 (the target location closest to the oscillatory focus; see Fig. 1B) and 4 other target positions (positions 2 and 3 in the same quadrant; position 6 in the opposite hemifield; and position 8 in the opposite quadrant of the same hemifield, see Fig. 1B). The observed phase differences were significant yet relatively small, only a fraction of a full oscillatory cycle. Since phase is a circular measurement (between 0 and 2π), however, any observed phase difference ‘Δϕ’ may be compatible with several actual values for the phase difference (for instance phases might be separated by less than one oscillatory cycle, i.e. phase difference = Δϕ; by 1–2 oscillatory cycles, i.e. phase difference = 2π + Δϕ; by 2–3 oscillatory cycles, i.e. phase difference = 4π + Δϕ, and so on). One way to disentangle these possibilities is to sample phase values at intervening points; if these values lie outside of the original Δϕ range, it will be clear that more than one cycle was originally spanned. In a second experiment, we thus investigated this question by increasing the spatial resolution of target locations within a single quadrant.

### Alpha oscillatory phase within a single quadrant spans no more than one cycle

**Figure 3:**
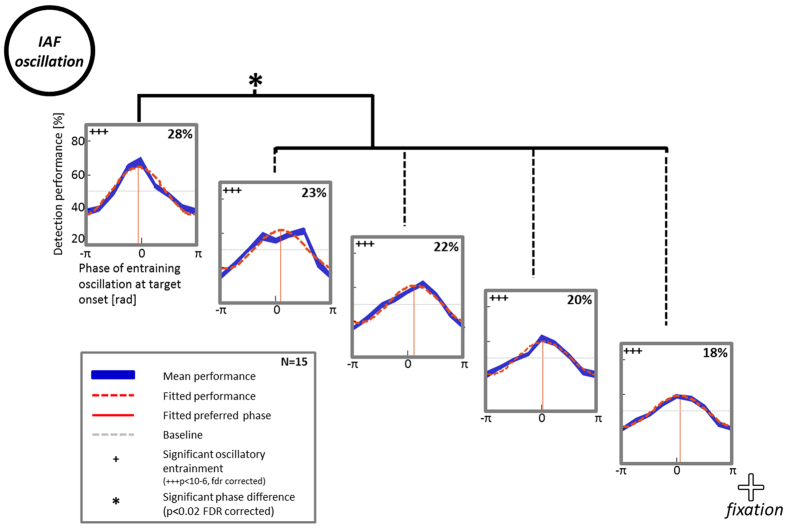
Phase differences at higher spatial resolution suggest phase differences within one oscillatory cycle. In Experiment 2, subjects performed the same task as in Experiment 1 but only 5 target locations were tested at a higher spatial resolution within a single quadrant, closest to the oscillatory focus (position 1 and 2 of Experiment 1 and three interleaved positions; drawing not to scale, see Fig. 1C). Detection performance was significantly entrained by the individual alpha frequency (IAF) luminance oscillation at all 5 target locations (orange dashed line: fitted data; in blue: mean performance ± sem over 15 subjects). Small but significant phase differences could be observed between position I (next to the disc) and all other positions.

In Experiment 2, we included target positions 1 and 2 of Experiment 1 (since oscillatory modulation of detection performance was strongest close to the disc) and interleaved 3 additional locations, logarithmically spaced. As for Experiment 1, we computed detection performance (hit rate) along the oscillatory phases for each subject and position separately. These data were fitted to a cosine function. A Monte Carlo test was performed on the averaged data (as in Experiment 1, see Methods), and revealed significant oscillatory entrainment of detection performance at all 5 tested target locations (22 ± 2% amplitude modulation on average; Fig. 3, dashed orange curve).

The motivation for this experiment was to increase the spatial resolution of target locations in order to assess whether the observed phase differences of Experiment 1 lay within one or multiple oscillatory cycles. If the small phase differences of Experiment 1 actually covered several oscillatory cycles, a closer distance between target locations might succeed in exposing larger phase differences. Comparing preferred phases for target detection between the 5 different target locations again revealed significant phase differences between position I (closest to the oscillatory focus) and all other positions (against positions II, and III: p < 10^–5^; against position IV: p < 0.01, against position V: p < 0.05). The magnitude of these phase differences, however, remained smaller than those obtained in Experiment 1. This suggests that all of the measured oscillatory phases (from both Experiment 1 and Experiment 2) likely lie within less than one oscillatory cycle of each other.

### Oscillatory entrainment of visual detection performance at different frequencies : 5Hz and 15Hz

Is entrainment of visual detection performance unique for the alpha rhythm or could other frequencies lead to a similar outcome? We investigated this question in Experiments 3 and 4, where we entrained at 5 and 15 Hz, respectively. Besides the frequency of the luminance oscillation, all other stimulus properties were similar to the original Experiment 2. 5 participants, who also took part in Experiment 2, performed these additional experiments.

**Figure 4:**
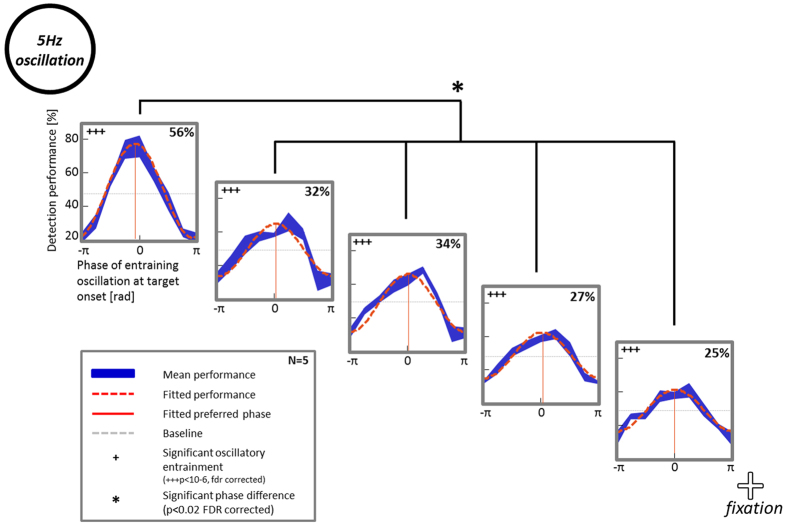
Oscillatory modulation of detection performance and significant phase differences with 5 Hz luminance oscillations. In Experiment 3, subjects performed the same task as in Experiment 2 but the disc’s luminance oscillated at 5 Hz instead of the individual alpha frequency of each subject. Detection performance was significantly entrained by the 5 Hz luminance oscillation at all 5 target locations (orange dashed line: fitted data; in blue: mean performance ± sem over 5 subjects). Just as for Experiment 2, small but significant phase differences could be observed between position I (next to the disc) and all other positions.

We analyzed the data of these two experiments in the same way as that of Experiment 2. We fitted detection performance at the different target positions to a cosine function and found that with an entrainment frequency of 5 Hz, detection performance was also following a significant oscillatory pattern at all 5 target positions (p < 10^–6^; Fig. 4). Moreover, the fitted amplitude averaged over the five participants was even higher than in Experiment 2 (two-way ANOVA, main effect of frequency, p < 2 × 10^–6^). This difference in amplitude was significant at positions 1 and 3 (post-hoc two sample t-tests; p < 0.01/0.04 (fdr corrected)). For both conditions, we observed an effect of target position on the amplitude of oscillatory performance fluctuations (two-way ANOVA, main effect of position, p < 0.0003). No interaction between factors frequency and target position was present. In addition, we found for Experiment 3 a significant difference between the preferred phase at position 1 and all other target positions, reminiscent of the pattern already observed in Experiment 2 (p < 0.01 (fdr corrected)). In fact, the phase differences between position 1 and all other positions were comparable across Experiments 2 and 3 (Parametric Watson-Williams multi-sample test for equal means, Matlab circular statistics toolbox^25^, p > 0.05).

**Figure 5:**
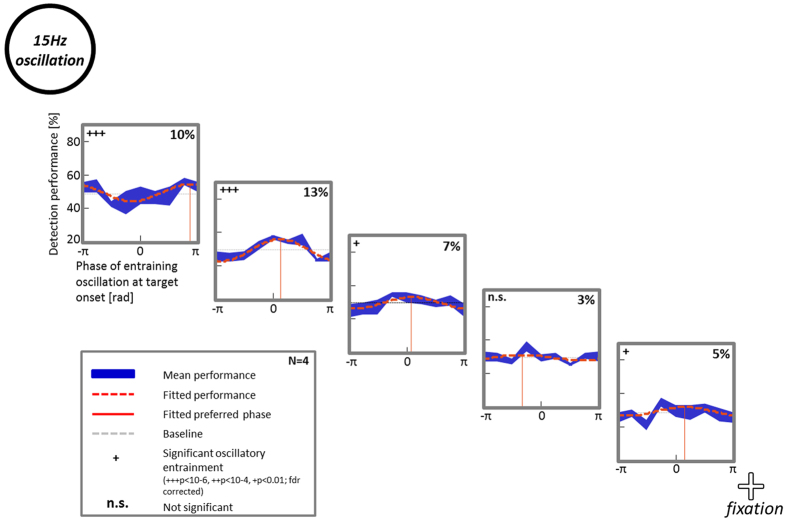
Oscillatory modulation of detection performance with 15 Hz luminance oscillations. In Experiment 4, subjects performed the same task as in Experiment 2 but the disc’s luminance oscillated at 15 Hz instead of the individual alpha frequency of each subject. Detection performance was significantly entrained by the 15 Hz luminance oscillation at 4 out of the 5 target locations (orange dashed line: fitted data; in blue: mean performance ± sem over 4 subjects), but no significant phase differences could be observed between different target positions.

With the peripheral disc oscillating at 15 Hz (Fig. 5), we found that detection performance showed a significant but much reduced oscillatory pattern at positions 1–4 (Monte-Carlo-Test, p < 10^–6^/p < 0.01 (fdr corrected)). The overall amplitude was significantly lower than that measured at alpha frequency in Experiment 2 (two-way ANOVA, main effect of frequency: p < 4 × 10^–7^). An effect of target position on the amplitude of performance modulations could again be observed (two-way ANOVA, main effect of position: p < 0.01). No interaction between frequency (alpha vs. 15 Hz) and target position was found. Moreover, with 15 Hz entrainment, no significant phase differences could be observed, presumably because the amplitude of sinusoidal modulations was too low to provide reliable phase estimates.

We compared the 3 different frequencies (5 Hz, IAF, and 15 Hz) in a two-way ANOVA. We found that oscillatory amplitude was significantly altered by frequency of the luminance oscillation (p < 4 × 10^–13^) and target position (p < 1 × 10^–5^). No interaction between factors was present. Two-sample t-tests revealed that the oscillatory amplitudes differed significantly between all conditions, with significantly higher values for lower frequencies than for higher frequencies (5 Hz vs. individual alpha: p < 0.01; 5 Hz vs. 15 Hz: p < 3 × 10^–4^; 15 Hz vs. individual alpha: p < 0.01).

### No oscillatory entrainment of detection performance for isolated flash events at different delays

Phase-related changes of target detection performance in Experiments 1–4 were interpreted as perceptual phase entrainment to the peripheral luminance oscillation. However, differing phase values also indicate that the target was presented earlier or later in time with respect to a highly salient visual event – the luminance highpoint in the modulation sequence of the large peripheral disc. It is conceivable that varying target latency around this highpoint could be sufficient to produce performance fluctuations similar to the ones we recorded, for example due to backward-masking (when the target appears before the luminance peak) or forward-masking mechanisms (when the target appears after the luminance peak). In this context, the difference in optimal phase across spatial positions in Experiments 1, 2 and 3 may be interpreted as changes of the optimal masking delay as a function of the target-mask spatial distance^26^,^27^,^28^. Importantly, this alternative interpretation does not involve oscillatory entrainment. This control experiment was designed to examine this possibility.

**Figure 6:**
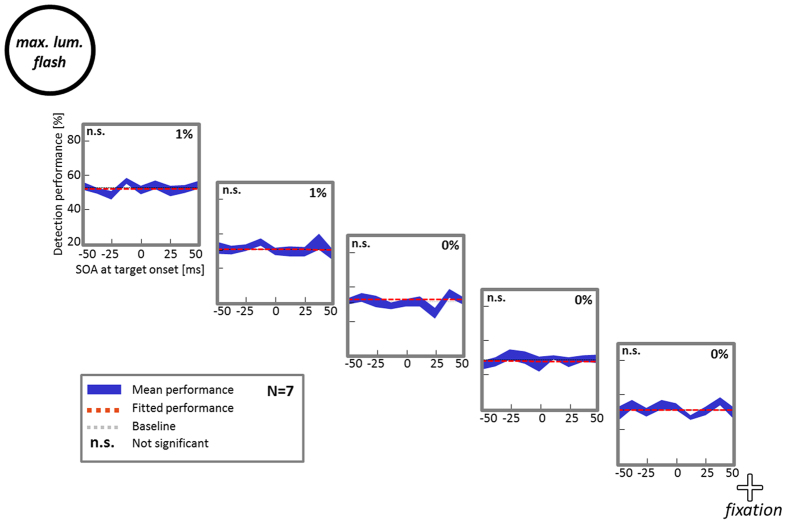
Control experiment rules out backward- and forward-masking as the cause of oscillatory perceptual entrainment. Subjects performed the same task as in Experiment 2, however the peripheral disc was not continuously oscillating but only flashing once (maximal luminance; *‘max.lum. flash’*) at different SOAs relative to target presentation (±50, 37.5, 25, 12.5, and 0 ms). No oscillatory pattern of detection performance was observed.

Here, we tested the same target locations as in Experiment 2–4 (Fig. 1C), but instead of presenting a continuously oscillating disc to entrain perceptual oscillations, the same disc was flashed (maximal luminance) for only one frame (6.25 ms). Brief targets (6.25 ms, as in the previous experiments) were presented at different SOAs before or after this flash (±50, 37.5, 25, 12.5 or 0 ms) to evaluate backward- and forward-masking, respectively. If masking by the peripheral luminance highpoint was responsible for the performance fluctuations observed in Experiments 1–4, we should expect similar performance fluctuations in this control experiment. As in Experiments 1–4, we calculated detection performance (hit rate) separately for each SOA and target location (Fig. 6, in blue) and fitted the data to a cosine function (Fig. 6, orange dashed curve). We applied the same Monte Carlo test to these data but the amplitude of detection performance fluctuations did not reach significance for any of the 5 tested positions (amplitude modulation averaged over positions: 0.5 ± 0.11%). This implies that the robust performance fluctuations observed in Experiments 1–3 (and to some extent, in Experiment 4) can safely be attributed to oscillatory phase entrainment of visual detection performance.

### Local oscillatory entrainment of detection performance for isolated flash events of different luminances

**Figure 7:**
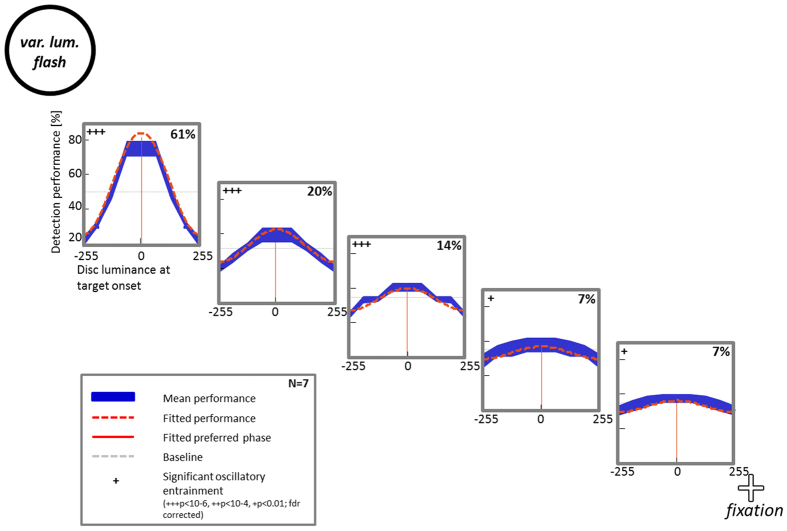
Influence of the disc’s luminance on sinusoidal modulation of detection performance. Subjects performed the same task as in Experiment 2, however the peripheral disc was not continuously oscillating but only flashing simultaneously with target presentation, at different luminance levels (*‘var. lum.* flash’, for *variable luminance flash*; 64, 128, 192, and 255). Detection performance was modulated in an oscillatory way by the disc’s luminance at all 5 target positions.

In a last control experiment, we tested whether detection performance merely follows the disc’s luminance, or whether the oscillatory modulation was truly the result of an entrained oscillation. Therefore, the peripheral disc was presented for only a single flash, simultaneously with the target stimulus, but using one of four different luminance levels (64, 128, 192, and 255), randomly assigned on each trial. These 4 luminance values were the same as those displayed at 7 of the 8 phase values of the disc’s luminance oscillation in Experiments 1–4. (The 8^th^ phase bin, corresponding to phase zero, could not be tested here since this would amount to flashing a black disc on a black background). We fitted detection performance data to a cosine function and extracted fitted amplitude values for each target position (Fig. 7). A Monte Carlo Test showed significant oscillatory modulation of detection performance at all 5 target locations (p = 10^–6^ for positions 1–3, p = 0.01 for positions 4 and 5; fdr-corrected).

We further compared the fitted sinusoidal modulation amplitudes measured in this control experiment with those obtained from Experiment 2, using a 2-way ANOVA with factors condition (control/original) and target position. This revealed that both conditions showed comparable amplitude values overall (no main effect of condition, p = 0.4); additionally, a decrease in amplitude across target positions could be observed for both conditions (main effect of position, p < 10^–4^). However, a strong interaction between the two factors (condition and target position) was found (p < 3 × 10^–8^). A post-hoc test on the spatial change of detection performance modulation amplitude indicated that the amplitude significantly decreased from position 1 (closest to the disc) to more distant positions for this control experiment (1 way ANOVA, p < 1 × 10^–8^; followed by a Tukey’s HSD multiple comparison test), but not for Experiment 2 (p > 0.05).

To conclude, detection performance can, under some conditions, follow the luminance fluctuations of a peripheral flash; this may account for some of the findings observed with oscillatory luminance modulation of the peripheral disc in Experiments 1–4. However, we note that this luminance contamination is a rather local effect (67% drop in magnitude observed between positions 1 and 2) whereas oscillatory entrainment of detection performance seems to spread over larger distances (e.g. 18% decrease in magnitude between positions 1 and 2 in Experiment 2). In addition, peripheral luminance at target onset cannot account for the entire pattern of results reported here, since the exact same luminance values produced markedly different performance fluctuations when the entraining frequency was varied from 5 Hz to alpha-frequency to 15 Hz (across Experiments 2–4).

## Discussion

Several studies revealed that the phase of ongoing alpha and theta (7–10 Hz) oscillations at the moment of presentation of a visual stimulus modulates its perception, such that opposite oscillatory phases can lead to opposed perceptual outcomes^5^,^6^,^7^,^8^. Thus, the phase of brain oscillations is an important factor in the processing of visual inputs. In at least certain situations, such as when a localized visual stimulus oscillates in luminance (like the peripheral disc in our experiments), brain oscillations can have a well-defined spatial origin in the cortex^12^. Indeed, due to the retinotopic organization of early visual areas, neurons responding to the oscillating visual stimulus are spatially adjacent in cortex. Their response will follow a specific activity pattern, oscillating at the same frequency as the entraining stimulus and with a constant phase relationship – a so-called ‘steady state visual-evoked potential’ (‘SSVEP’)^29^. But in such cases, what happens in more distant regions of the same retinotopic area? How does the oscillation, its amplitude and in particular its oscillatory phase, change as a function of spatial distance from the point of origin? Is the amplitude only significant within the neighborhood of the oscillatory source, or does it remain substantial also at distant locations? Does the phase synchronize across space^9^,^10^ or does it propagate as an oscillatory travelling wave?^19^,^20^,^12^,^13^,^14^ This is an important question since the remote phase will determine, in part, the likelihood that a distant visual stimulus will be perceived. Here we directly addressed this question in human observers by measuring the phase-dependence of visual detection at different distances from the oscillatory focus.

In Experiments 1 and 2, we found that target detection was significantly modulated by the phase of the entraining alpha oscillation at all tested locations, including remote locations (>9 degrees away) in opposite visual quadrants relative to the vertical or horizontal meridians. Moreover, in Experiments 3 and 4, we found that oscillatory modulation of detection performance can also be induced at other frequencies (5 and 15 Hz). When comparing different frequencies, a decrease in “oscillatory amplitude” of detection performance functions was observed with increasing frequency, which could be likened to the 1/f pattern of a classical EEG power spectrum^30^ –though of course, using only 3 oscillation frequencies, we cannot draw any direct conclusion about the entire shape of the spectrum of performance modulations across frequencies. Interestingly, the “optimal” phase of the entraining alpha-frequency and 5 Hz luminance oscillations was comparable at all target locations except the one closest to the entraining stimulus, which was significantly different. When entraining with 15 Hz, no such phase difference could be observed, possibly because oscillatory amplitude at that frequency was too weak to reliably determine the corresponding phase.

Although we did not record eye movements during the task, the importance of maintaining fixation throughout the experiment was explicitly emphasized to all subjects. In addition, it seems difficult to envision a scenario in which the entire pattern of results reported in our experiments would be an artifact of unwanted eye movements. While target detection can obviously be improved or impaired by moving the eyes closer or farther from a target, this could not explain the synchronized oscillatory fluctuations of detection performance across the entire visual field observed in Experiment 1: if the eyes had moved towards a given location and improved detection in that region of the visual field, this should also have come at the cost of decreased performance in opposite regions, and the corresponding performance fluctuations should have occurred in anti-phase. Another possibility may be that eye movement probability, rather than direction, was affected by the phase of the inducing luminance oscillation. For example, each luminance peak may have triggered a reflexive saccade, and the associated saccadic suppression^31^ may have affected detection performance across the entire visual field. However, even reflexive saccades have non-negligible latencies^32^ implying that the phase relation between saccade and luminance oscillation should be greatly affected by the frequency of the luminance oscillation: for a saccadic latency of 100 ms, for example, the peak-triggered saccade should occur at the next peak of an alpha luminance oscillation, but at the trough of a 5 Hz luminance oscillation. This explanation thus appears incompatible with the similar preferred phases for target detection observed at alpha-frequency in Experiments 1–2 and 5 Hz in Experiment 3. Overall, we thus consider an explanation of our findings solely based on eye movement artefacts unlikely.

Could it be that the oscillatory modulation of detection performance is only a signature of the sinusoidally increasing and decreasing luminance of the peripheral disc? We investigated this in a control experiment where the disc stimulus was briefly flashed simultaneously with the target stimulus, at different luminance levels. Detection performance was indeed strongly influenced by the disc’s luminance. However, the corresponding modulation amplitude decreased much faster across space relative to the experiments using genuine luminance oscillations. This suggests the possible existence of at least two independent entrainment effects, a local one in the neighbourhood of the oscillatory source, strongly affected by the luminance of the disc, and a more global one covering the majority of the visual field, and depending more on the actual oscillatory phase than on its associated luminance value. The phase differences repeatedly observed (in Experiments 1–3) between the target position closest to the disc and all other positions, which did not differ in phase from each other, also seem to support this distinction between a local and a more global entrainment effect of visual detection performance.

What may be the neural mechanisms supporting this oscillatory entrainment of detection performance? The local effect, besides its obvious contribution from the luminance of the oscillating disc, may also tentatively involve bottom-up processing (from retina to LGN to cortex) and well-established lateral connections within each cortical region^11^, producing an oscillation of neuronal excitability in line with the principle of an SSVEP^29^. On the other hand, the widely distributed pattern of oscillatory entrainment of detection performance (with little or no phase difference across space, but with a distinct phase from the “locally” produced one) can only be attributed to a global network mechanism involving widespread connectivity. Anatomically, the cortical representations of the upper- and lower-left quadrants are only contiguous in area V1, and their spatial separation increases along the hierarchy of cortical areas^33^; this makes it difficult to imagine that lateral connectivity alone could produce oscillatory behavior that would be uniformly phase-synchronized across such distances. Furthermore, the upper-left and upper-right quadrants are represented in distinct hemispheres for all visual cortical areas, and are only connected through callosal fibers^34^ as well as by common inputs from subcortical structures such as the thalamus^35^. In sum, it seems that the most plausible source for a common oscillatory modulation across 3 distinct visual quadrants is the subcortical route^36^; for example, certain thalamic nuclei such as the pulvinar have widespread connection patterns that can affect entire sensory areas either directly or via feedback through higher-level brain regions^37^,^38^,^39^. Because the pulvinar is a relatively small structure only a few millimeters wide in humans, it is easy to imagine that the localized luminance oscillation could result in a globally synchronized modulation of neural excitation across such a structure^36^, which would eventually result in a global phase entrainment of sensory perception across the visual field, as reported here. At positions near the oscillating disc, the superposition of this global effect and a local effect created through lateral connections (and/or luminance contamination) would lead to a significant difference of oscillatory phase relative to the rest of the visual field.

Recently, O’Connell and colleagues^40^ published a related finding in the auditory cortex of monkeys, when entraining neurons in A1 by presenting pure tone beeps with regular stimulus onset asynchronies (1.6 Hz). Auditory neurons that respond to stimuli of a specific frequency are arranged in the brain in a specific order, or in other words, tonotopically (much like visual neurons are organized retinotopically). O’Connell et al. showed that neurons that lie in the tonotopic region of a given stimulus entrain to the rhythmic stimulus at their high excitability phase, whereas neurons that lie outside of that tonotopic region, entrain at the opposite phase. If we consider that A1 neurons inside the target tonotopic region can be compared to visual neurons that lie in the retinotopic region of the peripheral oscillating disc in our experiment, and that auditory neurons outside of the tonotopic region correspond to neurons outside of the retinotopic region of the oscillating disc, then the findings of O’Connell et al. may represent an auditory equivalent to the phase difference we observed between position 1 (close to the peripheral disc) and all other positions.

In monkeys, the pulvinar has been shown to synchronize different brain regions during situations of intense attentional demands^41^. Therefore, it is possible that the relatively strong global effect observed in our experiments is directly linked to our task requirements, demanding that observers distribute attention in a sustained manner to the entire visual field (in Experiment 1) in order to detect targets appearing at random locations and moments in time. The influence of attention is also reflected in the difference in magnitude of oscillatory modulation of detection performance, which was about twice as large in Experiment 2 as in Experiment 1. We can tentatively attribute this difference to the more restricted range of target positions (all within 2.5 degrees of each other) in Experiment 2, allowing for a more spatially focused attention strategy. Indeed, it is often reported that oscillatory entrainment of brain activity (or SSVEP) increases with increasing attention^42^,^43^. Following this reasoning, one might predict that under different experimental conditions (with less attentional demands), the global modulation of detection performance may decrease in strength while the local effect would gain relative importance. Assuming that the local effect is produced not just by luminance contamination but also in part through lateral connections, decreasing attentional demands might then reveal a difference in preferred phases for detection performance for the different target locations, following the scheme of a travelling wave. In other words, such a travelling wave behavior might well be masked in our experiments by the overriding global effect. While retinotopic travelling waves have been demonstrated in several non-human species, evidence for travelling waves in humans is so far limited to propagating waves of activity between distinct cortical regions^44^,^45^,^46^,^47^,^48^, and this hypothesis thus remains merely speculative.

Finally, we observed a clear decrease in the overall amplitude of performance oscillations with increasing frequency of luminance oscillations. This decrease cannot be explained by an effect of attention, since task demands were similar for all conditions. Beyond a general tendency of brain oscillatory amplitudes to decline with increasing frequencies (as in the typical 1/f spectrum of the EEG), we believe that this change could also reflect the strength of the luminance contamination by the peripheral disc. The higher the frequency, the less time passes between distinct luminance values in the oscillatory signal; this means that each luminance value influences perception over a narrower and narrower time window. Ultimately the rapid succession of luminance values at high frequencies will conflate their effect on perception, abolishing the luminance contamination to oscillatory entrainment. In line with this idea, the amplitude difference between 5 Hz and alpha-frequency oscillatory entrainment of detection performance was strongest at the position closest to the disc, i.e. the position where the luminance contamination was maximal. Furthermore, at 15 Hz the performance oscillation at that same spatial position was dramatically decreased (7 times weaker than at 5 Hz, and 3.5 times weaker than at alpha frequency), even though the same luminance values had been presented at all frequencies.

In conclusion, our experiments reveal that oscillatory entrainment of visual behavior is not a strictly local phenomenon. Instead, a localized entraining oscillation in one part of the visual field modifies visual perception throughout the entire visual field. However, the phase of the entrained behavioral oscillation is not the same at distant locations as it is near the oscillatory source. This spatial pattern of oscillatory phases suggests the existence of a *local* entrainment mechanism near the source, directly influenced by the luminance of the peripheral disc, and a *global* entrainment that could be mediated by widespread connectivity from thalamic nuclei such as the pulvinar.

## Material and Methods

### Subjects

The experiment was approved by the *Comité de Protection des Personnes Sud-Ouest et Outre-Mer II* under protocol number 2013-A00544-41, and the methods were carried out in accordance with the approved guidelines. All subjects provided written informed consent before participating in this study. 10 volunteers (6 females, mean age: 27.6 ± 6.5 years) performed Experiment 1, where 9 target positions were tested. For the second experiment, where 5 target locations were tested, 15 volunteers (7 females, mean age: 26.9 ± 5.4 years) participated. 5 participants (30.6 ± 4.9 years) performed Experiments 3 and 4, using different entrainment frequencies. 8 further volunteers (3 females, mean age: 27.6 ± 4.3 years) performed a control experiment using a flashed peripheral stimulus (maximal luminance) without oscillatory entrainment of visual detection performance; here the data of one participant had to be excluded from analysis because of an error in the staircase procedure. Finally, 7 subjects (30.4 ± 4.6 years) participated in a further control experiment, where the effect of the disc’s luminance on detection performance was tested. One subject performed all 4 experiments, and 4 other subjects performed Experiments 2, 3, and 4.

Detection performance can be modulated periodically by entrained oscillatory activity^49^,^50^. Our interest was not in replicating these demonstrations, but rather in evaluating possible changes in the amplitude and phase of such entrained oscillations over space. We thus defined a criterion of subject selection prior to the experiment that included only those subjects in data analysis who showed a significant oscillatory modulation of detection performance (assessed as described below) for the target position closest to the oscillatory disc (position 1, Fig. 1B). With this criterion, 7 out of 10 subjects were included for analysis in Experiment 1, all 15 subjects for Experiment 2, all 5 participants of Experiment 3, and 4 out of 5 subjects for Experiment 4. In comparison, none of the subjects in the control experiment using a flashed disc at distinct SOAs emulating the different oscillatory phases would have passed this criterion (since no significant modulation was observed in that experiment). The corresponding distribution of positive and negative results for an oscillatory pattern of performance modulation (Experiment 1 and 2 using alpha oscillatory entrainment of detection performance: 22 vs. 3 observers; control experiment 1: 0 vs. 8 observers) was tested in a chi square test which revealed that our subject selection in the main (alpha-frequency) experiments was highly unlikely to occur merely from inter-subject variability in a task not designed to induce oscillatory behavior (p < 0.005).

### Stimuli

Stimuli were presented at 57 cm distance using a desktop computer (2.09 GHz Intel processor, Windows XP) with a cathode ray monitor (resolution: 640 × 480 pixels; refresh rate: 160 Hz) on a black background (luminance: 0.42 ± 0.02 cd/m^2^). Stimuli were designed and presented via the Psychophysics Toolbox^51^ running in MATLAB (MathWorks). The peripheral disc (radius: 1.75 degrees of visual angle; eccentricity: 7.5 degrees of visual angle) was oscillating sinusoidally between black (0.42 ± 0.02 cd/m^2^) and white (45.5 ± 2.7 cd/m^2^) at the individual alpha frequency of each subject, previously measured in a separate resting-state EEG experiment (mean frequency: Experiment 1: 9.57 ± 0.3 Hz sem; Experiment 2: 9.57 ± 0.35 Hz sem). In the first experiment, 9 target locations were chosen symmetrically in the two upper quadrants and in the lower left quadrant of the screen (3 locations in each quadrant; see Fig. 1B). Target locations in each quadrant were spaced according to cortical magnification (position 1/6/9: 5.4 degrees, position 2/5/8: 2.9 degrees and position 3/4/7: 1.5 degrees of eccentricity)^52^ to ensure equal spacing in the visual cortex. In the second experiment, only 5 target locations were tested in only the upper left quadrant (Fig. 1C). These 5 positions included two positions which were tested in Experiment 1 (positions 1 and 2, see Fig. 1B) and 3 positions in-between to provide higher spatial resolution. While the peripheral disc was oscillating at the individual alpha frequency of each subject, target stimuli appeared at 9 (Experiment 2: 5) possible target locations and 8 possible phase values of the luminance oscillation. In Experiments 3 and 4, we tested the effect of different entrainment frequencies (5 and 15 Hz), using the same paradigm and stimulus properties as in Experiment 2 (see Fig. 1C). One further control experiment investigated at the same 5 locations the effect of masking by the disc’s luminance, by presenting the peripheral disc for only one frame (6.25 ms) at maximal luminance at different stimulus-onset asynchronies (SOA = ± 50, 37.5, 25, 12.5 or 0 ms, covering one cycle of an average alpha oscillation of 10 Hz (100 ms) and corresponding to the phase values −∏, −3∏/4, −∏/2, −∏/4, 0, ∏/4, ∏/2, 3∏/4, ∏) preceding or following the target. In order to test whether detection performance follows the sinusoidal pattern of the entraining oscillation or only the disc’s luminance per se, we performed a final control experiment. Here, the peripheral disc was flashed for one frame, simultaneously with each target stimulus, at different luminance levels (64, 128, 192, and 255), corresponding to the different phase values of the disc’s luminance oscillation used in Experiments 1, 2, 3 and 4. In all experiments, 2 to 4 targets were presented for a single frame (6.25 ms) during each 6.25 s trial, at a randomly determined time but avoiding the first 800 and the last 800 ms, and leaving a minimal interval of 800 ms between targets. For each trial, the number of targets per trial was drawn from a decreasing probability distribution: 50% probability for 2 presented targets, 37.5% for 3 targets, and 12.5% for 4 targets. On average, 33 targets were presented for each subject and for each combination of phase and target position. Subjects were instructed to press a button within 800 ms of target detection. Target luminance was adapted, independently for each subject and target location, using a staircase procedure so as to ensure a detection rate of ~50%.

**Behavioral data analysis:** Based on its time of presentation, each target was assigned to 1 of 8 possible phase bins of the entraining luminance oscillation. For each oscillatory phase bin independently, detection performance was calculated as the percentage of correct trials (hit rate). The data was then fitted to a cosine function (with 3 free parameters: baseline level, amplitude, and phase) for each subject and target position separately.

In order to determine whether the fitted data showed significant oscillatory amplitude (and thus an oscillatory character) we performed a Monte Carlo test. Therefore, for each subject and target location separately, 50,000 virtual datasets were created by randomly assigning a performance value (correct/incorrect) to each trial recorded at each phase bin, under the null hypothesis that performance was comparable across phase bins (and thus equal to the average performance across phase bins). To determine the significance of an oscillatory performance fluctuation for a given subject and target location, we fitted corresponding performance curves from each of the 50,000 virtual datasets with a cosine function, and compared the cosine amplitude of the real data with the resulting surrogate distribution of cosine amplitudes. We obtained a p-value reflecting the proportion of surrogate cosine amplitudes that were equal to or higher than the experimentally observed cosine amplitude. To assess significance of oscillatory performance fluctuations across subjects at a given target location, we first averaged the data (real and virtual datasets) across subjects before fitting them with cosine functions, and compared the real and surrogate cosine amplitudes to obtain a p-value as before.

In a second step, the fitted cosine phases (phase at maximum amplitude) were compared pairwise between target locations (Experiment 1: 36; Experiment 2: 10 possible positions pairs). The significance of a given phase difference between two target locations was calculated using a Monte Carlo test. The null hypothesis was that both locations presented a performance oscillation, but with the *same* maximal phase. Therefore, performance data was first averaged across subjects, then across the two locations. Then, surrogate datasets were created for each position, taking into account the original distribution of trials across phase bins (calculated as the sum of trials of individual subjects for each phase bin); detection performance (correct/incorrect) for each trial was determined randomly based on average detection performance for the corresponding phase bin across the two target locations. Finally, for each of these surrogate position pairs, the phase difference was calculated. Resulting phase differences of surrogate datasets were then compared to the actual phase difference in the real data. P-values were calculated as the proportion of surrogate phase differences that were equal to or higher than the experimentally observed phase differences. Resulting p-values of phase differences were FDR-corrected to compensate for multiple comparisons. Since the statistical testing used here compared a grand-average measurement (e.g. average oscillatory amplitude across subjects) to a surrogate distribution of such grand-average measurements under the null hypothesis, it can be considered as ‘*fixed effect*’.

Data of Experiments 3 and 4, with 5 and 15 Hz as frequencies of the oscillatory disc, were analyzed in the exact same way as the data of Experiments 1 and 2. We further compared all conditions with oscillatory entrainment by the peripheral disc (5 Hz, individual alpha frequency, and 15 Hz) with each other across Experiments 2–4 (which probed the same spatial locations), using a Two-way ANOVA testing the effect of frequency and target position (1–5) on the obtained oscillatory amplitude values. Then, all possible comparisons between the different conditions (5 Hz vs. IAF; 5 Hz vs. 15 Hz; IAF vs. 15 Hz) were tested in a post-hoc two sample t-test. Additionally, we compared phase differences observed in Experiments 2 and 3 between position 1 and all other target positions using a circular one-way ANOVA^25^.

The first control experiment investigated the influence of masking by the disc’s luminance on detection performance at different SOAs. Here, we calculated for each subject and position separately the detection performance as percentage of correct trials (hit rate) across the different SOAs (±50, 37.5, 25, 12.25 and 0 ms). The resulting curve was then fitted to a cosine function and a Monte Carlo test was performed as described above (50,000 virtual datasets) to estimate significance of the “oscillatory” amplitude of detection performance. Since the Monte Carlo test revealed no significant oscillation at any of the tested target locations (and thus no preferred phase), the phase difference analysis was not performed on this dataset.

Data of the last control experiment, where the peripheral disc was flashed at different luminance levels (64, 128, 192 and 255), simultaneously with the target stimulus, was re-arranged into 8 “phase bins” to emulate an oscillatory pattern of luminance as follows: -∏, -3∏/4, -∏/2, -∏/4, 0, ∏/4, ∏/2, 3∏/4, were respectively assigned the performance obtained at luminance 255, 192, 128, 64, 64, 64, 128, 192 (each luminance was repeated, as would happen within one oscillatory cycle; since a 0-luminance flash could not be presented on the black background, we approximated the corresponding performance by its next neighbour, 64). The resulting performance function was fitted to a cosine function in order to reveal its “oscillatory” character. The fitted amplitude values were extracted and later, in a Monte Carlo test, compared to fitted amplitudes of 50,000 surrogate datasets in order to compute significance of the oscillatory pattern at each target position. Because of the symmetric construction of the data curves, preferred phases were restricted to 0 or pi; thus, we did not perform a phase analysis for this experiment. We further compared the results of this control experiment with those of Experiment 2 by using a two-way ANOVA investigating the influence of condition (IAF luminance oscillation vs. single flash at different luminance levels) and target position on the obtained fitted amplitude values. A post-hoc two sample t-test then compared the amplitude values of the conditions for each target position.

## Additional Information

**How to cite this article**: Sokoliuk, R. and VanRullen, R. Global and local oscillatory entrainment of visual behavior across retinotopic space. *Sci. Rep.* **6**, 25132; doi: 10.1038/srep25132 (2016).
